# Histone Deacetylase Complexes Promote Trinucleotide Repeat Expansions

**DOI:** 10.1371/journal.pbio.1001257

**Published:** 2012-02-21

**Authors:** Kim Debacker, Aisling Frizzell, Olive Gleeson, Lucy Kirkham-McCarthy, Tony Mertz, Robert S. Lahue

**Affiliations:** Centre for Chromosome Biology, School of Natural Sciences, National University of Ireland Galway, Galway, Ireland; University of Washington, United States of America

## Abstract

Expansions of DNA trinucleotide repeats cause at least 17 inherited neurodegenerative diseases, such as Huntington's disease. Expansions can occur at frequencies approaching 100% in affected families and in transgenic mice, suggesting that specific cellular proteins actively promote (favor) expansions. The inference is that expansions arise due to the presence of these promoting proteins, not their absence, and that interfering with these proteins can suppress expansions. The goal of this study was to identify novel factors that promote expansions. We discovered that specific histone deacetylase complexes (HDACs) promote CTG•CAG repeat expansions in budding yeast and human cells. Mutation or inhibition of yeast Rpd3L or Hda1 suppressed up to 90% of expansions. In cultured human astrocytes, expansions were suppressed by 75% upon inhibition or knockdown of HDAC3, whereas siRNA against the histone acetyltransferases CBP/p300 stimulated expansions. Genetic and molecular analysis both indicated that HDACs act at a distance from the triplet repeat to promote expansions. Expansion assays with nuclease mutants indicated that Sae2 is one of the relevant factors regulated by Rpd3L and Hda1. The causal relationship between HDACs and expansions indicates that HDACs can promote mutagenesis at some DNA sequences. This relationship further implies that HDAC3 inhibitors being tested for relief of expansion-associated gene silencing may also suppress somatic expansions that contribute to disease progression.

## Abstract 2

Genetic analysis in budding yeast and in cultured human astrocytes reveals that specific histone deacetylase complexes accelerate expansion mutations in DNA triplet repeats.

## Author Summary

The human genome contains numerous DNA trinucleotide repeats, which mutate infrequently in most situations. However, in families affected by certain inherited neurological diseases such as Huntington's, a trinucleotide repeat has undergone an expansion mutation that lengthens the repeat tract. This expansion is generally sufficient to cause disease. Further germline and somatic expansions in affected families occur at very high frequencies—approaching 100% in some cases—suggesting that mutation of the trinucleotide repeat becomes the norm rather than the exception, while the rest of the genome remains genetically stable. These observations indicate that trinucleotide repeat expansions are localized in the genome and occur by novel mutational mechanisms. We searched for proteins that favor expansions and identified specific histone deacetylase complexes (HDACs)—comprising enzymes that remove acetyl groups from histones—in budding yeast and in human astrocytes. Interfering with these HDACs by mutation, RNA interference, or small molecule inhibitors blocked 50%–90% of expansion events. We also found that yeast HDACs promote expansions via a downstream deacetylation target, the nuclease Sae2. These results indicate that HDACs promote trinucleotide repeat expansions by modulating key proteins, which in turn catalyze the expansion. We postulate that HDAC inhibitors, currently being tested for relief of the transcription-related consequences of expansions, may have the beneficial side effect of reducing the risk of further somatic expansion.

## Introduction

The relentless expansion of trinucleotide repeats (TNRs) causes Huntington's disease (HD), myotonic dystrophy type 1 (DM1), and at least 15 other inherited neurological disorders [1]. It is thought that expansions are actively promoted by the presence of key proteins, not their absence, probably due to the “corruption” of their normal biochemical activities by TNR DNA [2]–[4]. Evidence for promoting factors includes the fact that disease alleles expand at high frequencies, sometimes approaching 100% [5], in otherwise normal individuals and in a number of transgenic and knockin mouse models of HD and DM1 [6]–[12]. Using candidate gene approaches, the DNA repair factors Msh2, Msh3, Pms2, Ogg1, and Xpa were identified as promoting proteins in mice, based on the fact that somatic expansions are suppressed ∼50%–90% by homozygous knockout of *Msh2*, *Msh3*, *Pms2*, *Ogg1*, or *Xpa* [6]–[13]. Knockout of *Msh2* or *Msh3* also largely eliminates intergenerational expansions [7],[9],[10],[14]. Thus, key DNA repair components promote expansions in certain mouse models.

The transgenic mice studies described above monitor long, disease-causing TNRs becoming even longer. For example, commonly used HD mouse models carry CAG tracts of 110–120 repeats [10],[12]. A human inheriting an HD allele in this length range would develop the disease as a young child [15]. As an alternative approach, we focus on expansions near the crucial threshold, a narrow range of allele lengths (∼30–40 uninterrupted repeats in humans [2],[4],[16]) that demarcates stable shorter repeats from unstable longer tracts. Expansion risk in humans and in yeast increases sharply once the threshold is crossed [17],[18]. Expansions crossing the threshold are critical initiating mutations leading to enhanced instability and disease [2]–[4]. It is not known whether the mechanism of expansion is the same for threshold-length alleles and long, disease-causing tracts. In this study, we find that yeast mutants lacking the nucleases Sae2 or Mre11 reduce expansion rates for (CTG)_20_ alleles, whereas *sae2* or *mre11* mutants show increased expansion frequencies for long (CAG)_70_ repeats [19]. This new evidence suggests that triplet repeat length helps determine expansion mechanism.

The goal of this study was to identify novel factors in yeast and human cells that promote expansions of TNR alleles near the threshold. We found specific histone deacetylase complexes (HDACs) that promote expansions, plus one human histone acetyltransferase (HAT) that inhibits expansions, and we suggest a mechanistic link between HDACs and DNA repair. These results indicate a causal relationship between HDACs and expansions, and they show that protein acetylation and deacetylation are key modulators of TNR instability.

## Results

### Yeast HDACs Rpd3L and Hda1 Promote CTG•CAG Repeat Expansions

**Figure 1:**
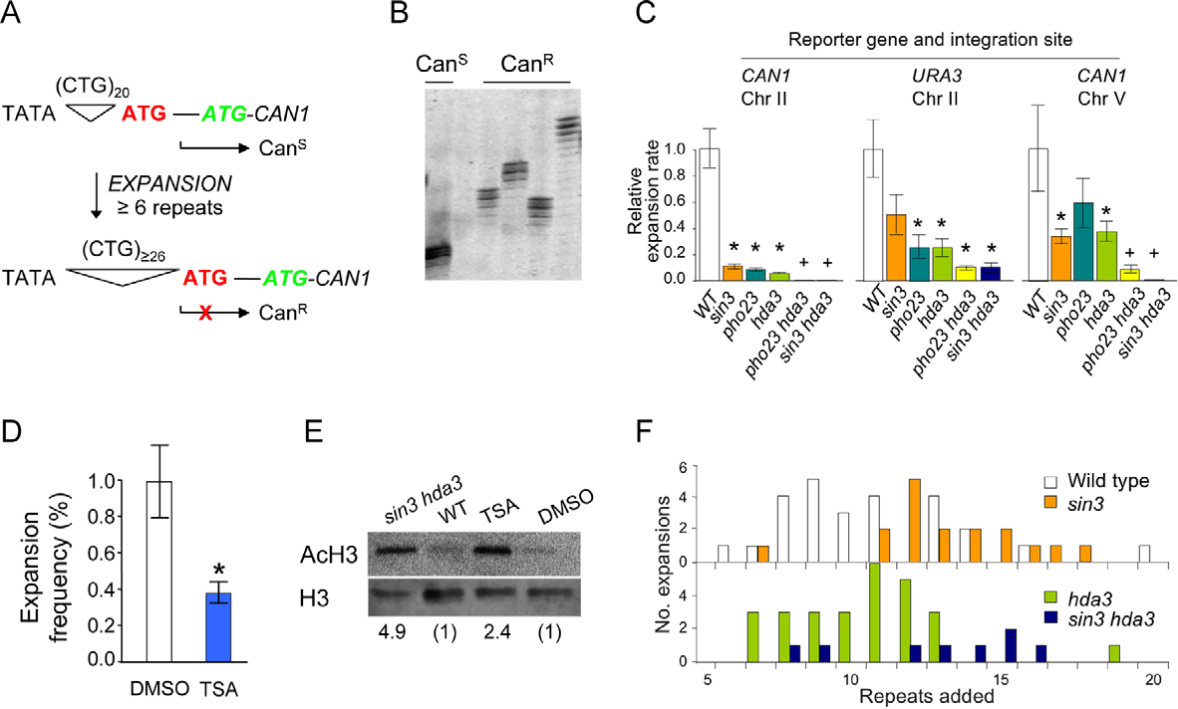
Mutation or chemical inhibition of yeast HDACs suppresses TNR expansions. (A) Reporter with (CTG)_20_ permits expression of the reporter gene *CAN1*, and results in canavanine sensitivity. Expansions of ≥6 repeats alter transcription initiation, incorporating the out-of-frame ATG codon that blocks expression of *CAN1* (X). Canavanine resistance ensues. (B) PCR products displayed on a high-resolution polyacrylamide gel. All expansion results reported here include PCR validation. (C) Expansion rates in mutants of Rpd3L (*sin3* or *pho23*), Hda1 (*hda3*), or both (*pho23 hda3* or *sin3 hda3*). TNR reporter integration sites are indicated in the figure. Error bars, ±SEM; * *p*<0.05 compared to wild type; + *p*<0.05 compared to wild type and to each single mutant (details in Table S1). (D) Cells were grown 13–14 generations in liquid culture ±30 µg/ml TSA, followed by expansion analysis. Error bar, ± SEM; * *p* = 0.02 compared to DMSO-only control, *n* = 5 independent measurements. (E) Accumulation of acetylated histone H3 in yeast cells with impaired HDAC activity. Immunoblot results of 15 µg protein from whole cell lysates. Top, acetylated H3; bottom, total H3. Values below the blot show the ratio of acetylated H3/total H3. (F) Expansion sizes, derived from PCR analysis. 26 genetically independent expansions for wild type, 17 for *sin3*, 25 for *hda3*, and 8 for *hda3 sin3*.

If specific proteins promote TNR expansions, then mutants deficient in these proteins will have fewer expansions. A large-scale yeast mutant screen was performed to identify mutants with reduced expansion rates. Cells with a (CTG)_20_-*CAN1* reporter (Figure 1A) were randomly mutagenized with a disruption library. A (CTG)_20_ repeat tract was utilized, as this allele length is near the apparent threshold in yeast [18]. Reduced expansion rates are manifested as fewer canavanine resistant cells (Figure S1). Nine thousand disruptants, covering approximately 50% of non-essential genes, were subjected to several rounds of screening with increasing stringency. Eleven mutant genes were identified that consistently suppressed TNR expansions (Figure S1). Three of the 11 genes were *SIN3*, *PHO23*, and *HDA3*. *SIN3* encodes a subunit of histone deacetylases Rpd3L and Rpd3S, whereas the subunit encoded by *PHO23* is unique to Rpd3L. *HDA3* encodes a subunit of another HDAC, Hda1. The *hda3* mutant was found twice, along with single isolates of *sin3* and *pho23*. Thus, a blind screen pulled out three genes encoding components of Rpd3L and Hda1, an enrichment of ∼100-fold compared to random chance. This clustering of mutations in related enzymes suggested a causal relationship between specific HDACs and TNR expansion.

Targeted knockouts of *sin3*, *pho23*, and *hda3* confirmed the gene assignments and allowed further analysis of expansions. Expansion rates were quantified using two reporters, *CAN1* (Figure 1A) and *URA3* [18], and all expansions were confirmed by PCR (Figure 1B). If an HDAC mutant primarily affects the instability at the triplet repeat, independently of the readout gene, then similar phenotypes would be expected for assays with *CAN1* and *URA3*. This outcome was observed (Figure 1C and Table S1). Single mutants of *sin3*, *pho23*, and *hda3* showed 9- to 18-fold reductions in expansion rates for the *CAN1* reporter integrated into chromosome II (Figure 1C, left panel). Expansion rates were reduced >1,000-fold in the double mutants *pho23 hda3* and *sin3 hda3*, which are simultaneously deficient in both Rpd3L and Hda1. When the reporter gene was *URA3*, a similar pattern of suppressed expansion rates occurred (Figure 1C, middle panel). The magnitude of the phenotype was somewhat smaller: 2- to 4-fold suppression in expansion rates for single HDAC mutants, and 10- to 18-fold for the double mutants. Thus, both *CAN1* and *URA3* reporters integrated at the same locus yielded similar outcomes, suggesting that Rpd3L and Hda1 affect instability of the TNR. To exclude a position effect, the *CAN1* reporter was relocated to an integration site on chromosome V. Suppression of expansions was again seen for the HDAC mutants (Figure 1C, right panel). Single mutants reduced expansion rates by 2- to 3-fold, while the *pho23 hda3* and *sin3 hda3* double mutants yielded 12- to 340-fold effects. In total (Figure 1C), the single mutants *sin3*, *pho23*, or *hda3* showed significant reduction in CTG expansion rates in seven of nine assays. All six assays using the double mutants, *pho23 hda3* or *sin3 hda3*, consistently gave lower expansion rates, and the double mutant effect was always stronger than for the single mutants. HDAC mutants in a common commercial strain, BY4741, also displayed reduced expansion rates for *CAN1* integrated at *LYS2*. Relative to wild type, expansion rates in the *sin3* mutant were strongly suppressed (>100-fold), with a milder phenotype for *pho23* (3-fold reduced), and a small but not statistically significant reduction of 1.7-fold for *hda3*. Overall, targeted knockout of Rpd3L and/or Hda1 suppressed expansion rates in most assays, and expansions were almost completely eliminated in some cases.

Expansion suppression could be phenocopied by treating wild type cells with trichostatin A (TSA), which inhibits many but not all HDACs [20]. TSA reduced expansion frequencies by 2.6-fold (Figure 1D) at a concentration that inhibits most HDAC activity of Rpd3 and Hda1 in vitro [21]. This finding is consistent with a published report showing that TSA-treated *Drosophila* had ∼3-fold fewer expansions of a (CAG)_78_ transgene, with preferential modulation of +1 repeat changes relative to other sizes [22]. In yeast, expansion sizes were similar with or without TSA, ranging from +6 to +19 repeats (Figure S2). Cells with impaired HDAC function showed the anticipated accumulation of acetylated histone H3, by nearly 5-fold in the *sin3 hda3* mutant and about 2.4-fold in wild type cells treated with TSA (Figure 1E). Compared to the HDAC mutants, TSA gave smaller effects on both expansion levels and the accumulation of acetylated histone H3, presumably due to incomplete inhibition by the drug.

Several control experiments eliminated trivial explanations of the HDAC effect on expansions. The range of expansion sizes was similar in wild type cells, HDAC mutants, and TSA-treated cells (Figures 1F and S2), indicating that HDAC status did not affect the genetic selection for expansions. Rather, the expansion size data suggest that HDACs likely govern initiation of expansions; there are fewer initiation events when HDACs are mutated or inhibited, but once the process is started the final size of the expansion is similar. There was no growth disadvantage of the HDAC mutants, with or without an expanded TNR, under conditions that select for expansions (Figures S3 and S4). *CAN1* transcript levels varied by 2-fold or less in the HDAC mutants (Table S2), showing no correlation with changes in expansion rates. Finally, suppression of expansions was primarily attributable to Rpd3L and Hda1, because only modest expansion phenotypes occurred in mutants defective in the alternative HDACs Rpd3S, Hos1, Hos2, Hos3, or Sir2 (Figure S5). In summary, mutation or chemical inhibition of yeast Rpd3L and Hda1 suppresses CTG repeat expansions by 50%–90%, with even greater effects in some mutant strains. These data support a mechanistic link between triplet repeat expansions and the yeast HDACs Rpd3L and Hda1.

### Human HDAC3, a Homolog of Yeast Rpd3L, Promotes Expansions in Cultured Human Astrocytes

**Figure 2:**
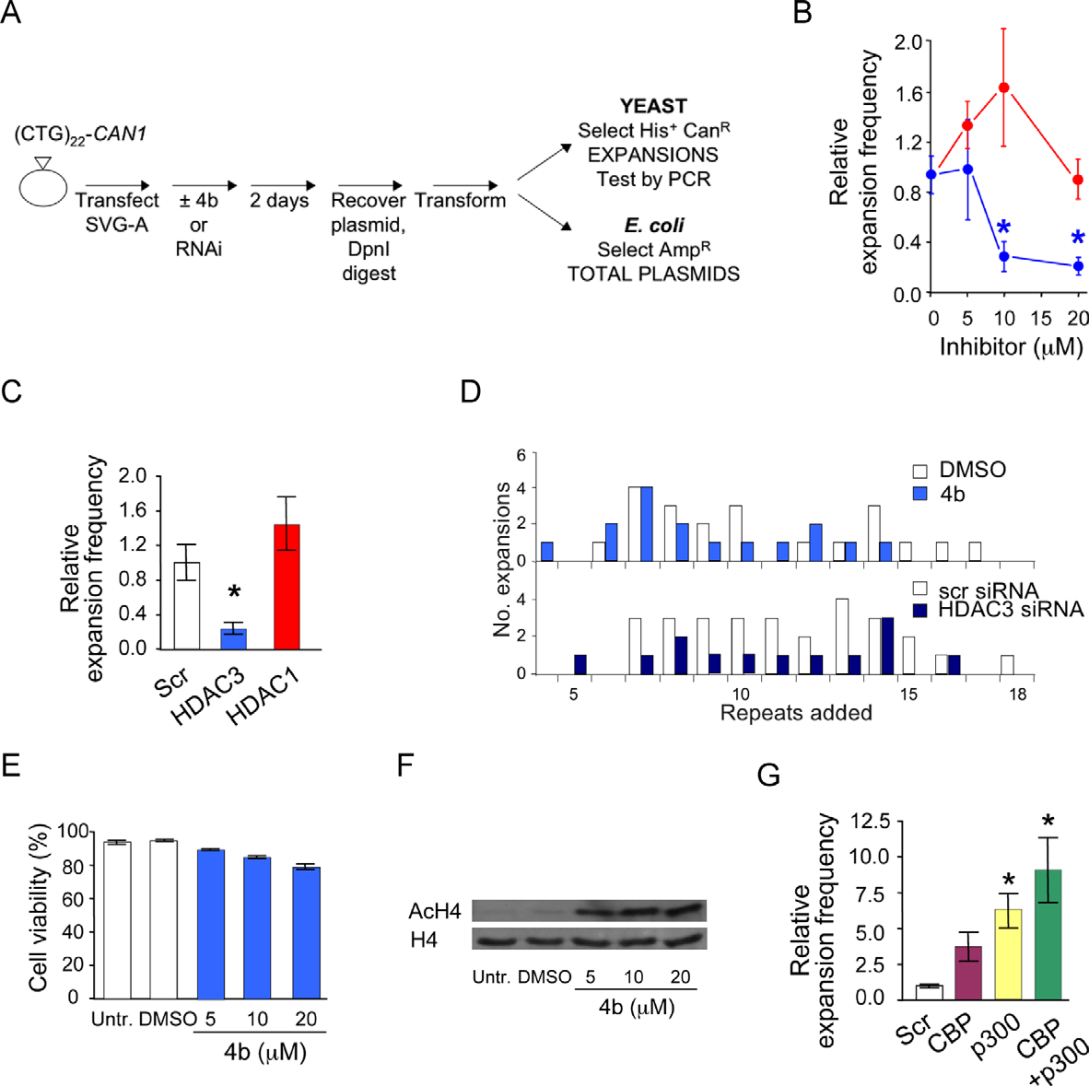
Chemical inhibition or RNAi knockdown of HDAC3 in human SVG-A cells suppresses expansions. (A) The genetic assay is essentially as described [27]. Cells were treated with either HDAC inhibitor **4b**, compound **3**, or DMSO only. Alternatively, siRNA was used with scrambled siRNA as a control. Expansions are scored using yeast as a biosensor, and total plasmid counts are monitored by bacterial transformation for enhanced sensitivity. (B) Expansion frequencies as a function of inhibitor dose, compared to DMSO-treated control cells. Blue, **4b**-treated; red, compound **3**-treated. Error bar, ±SEM; * *p*<0.05 compared to DMSO-treated cells. Details in Table S3. (C) Expansion frequency after RNAi. Knockdown efficiency, judged by three independent immunoblots, averaged 76(±8)% for HDAC3 and 76(±2)% for HDAC1. Error bars, ±SEM; * *p*<0.05 compared to scrambled control. Details in Table S3. (D) Expansion sizes, derived from PCR analysis. 21 genetically independent expansions for DMSO, 16 for **4b** (combined data from 10 µM and 20 µM treatments), 28 for scrambled siRNA, and 13 for HDAC3 siRNA. (E) Cell viability measured by nigrosin staining just prior to cell harvest. (F) Representative immunoblot of acetylated histone H4 and total histone H4 upon treatment with **4b**; data summary in Figure S6. (G) Expansion frequencies after RNAi against histone acetyltransferases. Error bars, ±SEM; * *p*<0.05 compared to scrambled control.

To address whether HDACs promote expansions in human cells, we focused on class I human HDACs, the homologs of yeast Rpd3 [23]. The small molecule inhibitor **4b** is selective for the class I enzyme HDAC3 but with some activity against HDAC1 [24]. **4b** treatment reverses *FXN* gene silencing in primary cells from Friedreich's ataxia patients [24] and relieves disease phenotype and transcriptional abnormalities in HD transgenic mice [25]. In light of the yeast experiments presented above, we posited that HDAC inhibition by **4b** might have the added benefit of suppressing expansions in human cells. To test this idea, CTG repeat expansions were measured in a cultured human astrocyte cell line, SVG-A. Glial cells such as astrocytes show somatic expansions in HD patients [26], and SVG-A cells support expansions in culture, as measured by the assay shown in Figure 2A [27].

**4b** efficiently suppresses TNR expansions in SVG-A cells at doses that are well tolerated. Treatment with **4b** reduced expansion frequencies in a dose-dependent manner (Figure 2B and Table S3). Compared to the DMSO-only control, expansion frequencies were suppressed 70% and 77% by **4b** at 10 µM and 20 µM, respectively. In contrast, treatment of SVG-A cells with an HDAC1- and HDAC2-selective inhibitor called compound **3** [28] did not suppress expansion frequencies (Figure 2B; small increases were not significant). Together, the inhibitor results suggest HDAC3 is the relevant target. Confirmation came from RNAi knockdowns. Knockdown of HDAC3 resulted in 76% reduction in expansion frequencies (Figure 2C), the same extent seen at the highest doses of **4b**, whereas knockdown of HDAC1 elevated the expansion frequency slightly but not to a statistically significant level. Inhibiting HDAC3 with **4b** or knocking it down changed the frequency of expansions, not their sizes (Figure 2D). Expansions added as many as 18 repeats to a starting tract of 22 repeats; thus, some expansions regulated by HDAC3 in SVG-A cells cross the threshold of 30–40 repeats observed in humans [2],[4],[16]. The reduced number of expansions upon **4b** treatment could not be attributed to increased cell death, because the SVG-A cells retained ≥83% viability, relative to DMSO-only control, even at the highest dose of inhibitor (Figure 2E). Molecular analysis of global histone H4 acetylation showed the anticipated increase in acetylated H4, up to about 10-fold, when cells were treated with **4b** (Figures 2F and S6). The opposite phenotype—increased expansions—was seen with RNAi knockdown of the histone acetyltransferases CREB-binding protein (CBP) and p300 (Figure 2G), consistent with observations in *Drosophila* [22]. We conclude that HDAC3 and CBP/p300 have opposing effects on expansions in SVG-A cells, with HDAC3 promoting TNR expansions.

### Rpd3L and Hda1 Promote Expansions in Trans, Partly through Sae2

**Figure 3:**
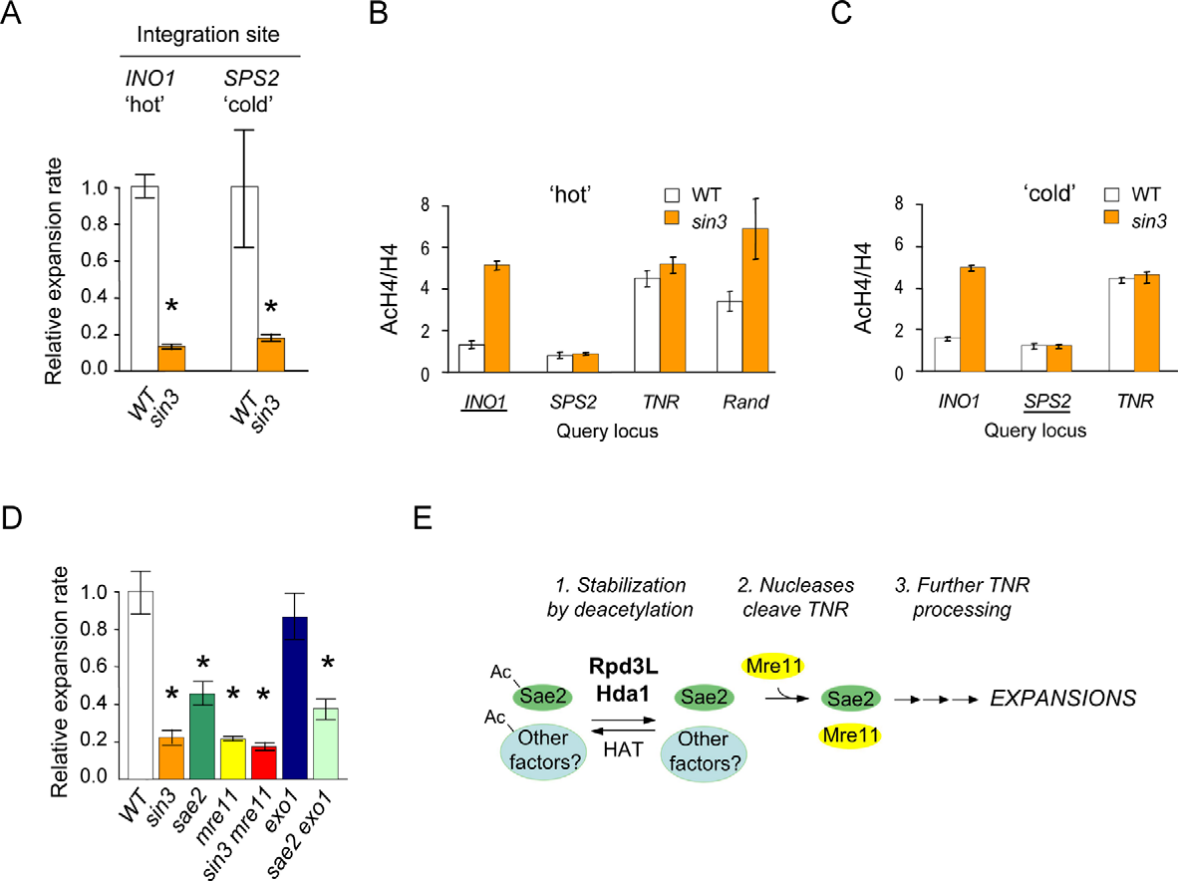
Evidence that Rpd3L acts in trans to promote expansions. (A) *sin3* mutants suppress expansion rates when the TNR reporter is integrated at “hot” zone, *INO1* on chromosome X and a “cold” zone, *SPS2* on chromosome IV. Error bars, ±SEM; * *p*<0.05 compared to wild type. (B, C) Chromatin immunoprecipitation using antibodies against pan-acetylated histone H4 or total H4. Underline indicates the TNR reporter integration site at *INO1* (B) or *SPS2* (C). Rand, control reporter with randomized sequence in place of triplet repeat. Error bars, ±SEM. Primer site details are provided in Figure S8. (D) Expansion rates in single or double mutants of *sae2*, *mre11*, *exo1*, and/or *sin3*. The reporter was (CTG)_20_-*CAN1* integrated on chromosome II. Error bars, ±SEM; * *p*<0.05 compared to wild type. Details for panels (A–D) are in Table S4. (E) Model for HDAC promotion of expansions in yeast. 1. Acetylated Sae2 (Ac-Sae2) is marked for degradation, but it is stabilized by deacetylation in an Rpd3L- and Hda1-dependent manner [32]. The same HDACs may deacetylate other factors relevant to expansions, thereby stabilizing them or influencing their activities. The action of Rpd3L and Hda1 is counterbalanced by one or more HATs that await identification. 2. Sae2 along with another nuclease, Mre11, cleaves TNR DNA, possibly in a hairpin structure, to initiate the expansion pathway. 3. The cleaved TNR undergoes additional processing steps to complete the expansion.

We first tested the idea that expansion rates are suppressed in cis by hyperacetylation of histones near the repeat tract, as might occur in HDAC mutants. The approach took advantage of previous studies showing that transcription and histone acetylation at some yeast genes are particularly sensitive to the absence of *SIN3*. One such locus is the *INO1* gene, which we refer to as a “hot” zone. In *sin3* mutants compared to wild type, transcript levels increase about 30-fold [20],[29] and histone acetylation increases 3.6- to 5-fold [30],[31] at *INO1*. If expansions are sensitive to local histone acetylation, then integration of the TNR reporter at *INO1* should give an enhanced *sin3* phenotype, i.e. show greater suppression of expansions. Similarly, there should be less *sin3* phenotype on expansions at a “cold” zone like *SPS2* whose expression and histone acetylation is nearly unaffected in a *sin3* mutant [20],[29],[30]. The results indicate otherwise (Figure 3A). For both integration sites, hot and cold, the effect of *sin3* on expansions was similar (6.4-fold suppression at *INO1*, 5.7-fold at *SPS2*). Nearly identical suppression effects were seen when the reporter was integrated at another relatively cold locus, *LYS2* (8.8-fold; Figure 1C, left panel), or at another hot zone locus, *IME2* (8.8-fold; unpublished data).

Confirmation studies of chromatin acetylation at the TNR locus led to an unanticipated result. Chromatin immunoprecipitation (ChIP) was used to evaluate pan-acetylation of histone H4 compared to total H4 at *INO1*, *SPS2*, and the TNR reporter (Figure 3B and C). H4 acetylation at *INO1* was increased 3- to 5-fold in the *sin3* mutant as expected for a hot zone, while H4 acetylation at *SPS2* was low in both the wild type and *sin3* strains, typical of a cold zone. These findings are independent of the integration site of the TNR reporter (compare Figure 3B and 3C), indicating that insertion of the reporter does not alter acetylation levels at either integration locus. Unexpectedly, we found that histones near the TNR are hyperacetylated, regardless of *SIN3* status, to about the same level as *INO1* in the *sin3* mutant (Figure 3B and C). Hyperacetylation seems to be conferred in part by the trinucleotide repeat, because a control reporter with a randomized sequence in lieu of the TNR yielded a greater dependence of histone acetylation on *SIN3* status (“Rand,” Figure 3B). Although the TNR is not uniquely responsible for hyperacetylation of nearby histones (Figure S7), it does contribute.

We concluded from the results in Figure 3A–C that HDACs most likely promote expansions in trans, perhaps by controlling the expression or stability of factors that expand the TNR. The nuclease Sae2 was investigated because a recent study showed Sae2 is stabilized by deacetylation in an Rpd3- and Hda1-dependent manner [32]. Furthermore, Sae2, along with the Mre11/Rad50/Xrs2 complex, is known to process hairpin DNA in vivo and in vitro [33],[34]. Since TNR expansions are thought to involve structured intermediates such as a hairpin [2]–[4], we tested the idea that an *sae2* mutant would suppress expansions. The *sae2* mutant partially suppressed expansions when compared side-by-side with a *sin3* mutant (Figure 3D), consistent with the idea that Sae2 is one (but not the sole) relevant target of Rpd3. Mutation of the nuclease encoded by *MRE11* suppressed expansions as much as the *sin3* mutant (Figure 3D). Although Rpd3 is not known to directly regulate Mre11, the expansion phenotype of the *mre11* mutant is consistent with the possibility that HDACs stabilize Sae2, which then works together with Mre11 to promote expansions. In support of this idea, the expansion phenotype of the *sin3 mre11* double mutant was indistinguishable from those of the *sin3* and *mre11* single mutants (Figure 3D). In contrast, loss of the Exo1 exonuclease showed no effect on expansions, and the *sae2 exo1* double mutant was no more defective than the *sae2* single mutant (Figure 3D). Together, the results of Figure 3 suggest that yeast Rpd3L and Hda1 promote expansions in trans through the nucleases Sae2 and Mre11.

## Discussion

This study reveals that yeast Rpd3L and Hda1 and human HDAC3 promote expansions of threshold-length triplet repeats in budding yeast and human astrocytes. Interfering with HDAC function through mutation, RNAi knockdown, or small molecule inhibitors eliminates most expansions. It is striking that yeast Rpd3 and Hda1 elicit opposite effects on genetic stability depending on the genomic context; these HDACs accelerate mutagenesis at triplet repeats, whereas they favor chromosome stability via the DNA damage response and processing of double strand breaks [32]. We also found that the human HATs encoded by CBP and p300 have the contravening effect of stabilizing triplet repeats. The latter finding complements an earlier report that CBP modulates instability of long repeats in *Drosophila* [22]. The relevant yeast HAT remains to be identified. The identification of HDACs as promoting factors and the protective action of HATs emphasizes the importance of protein acetylation/deacetylation to expansions. The mechanistic and therapeutic implications of these findings are considered below.

As in double strand break processing [32], one downstream target of Rpd3L and Hda1 is likely to be the nuclease Sae2. We propose a model where Rpd3L and Hda1 positively regulate Sae2 by stabilizing it. Sae2 and Mre11 then function together as nucleases to promote expansions (Figure 3E). This model is based in part on the study of Robert et al., who found that acetylated Sae2 is degraded by autophagy, but that Sae2 is stabilized by deacetylation in an Rpd3- and Hda1-dependent manner [32]. Also consistent with the Robert et al. work, we infer that Sae2 is not the only relevant target of these HDACs because the expansion phenotype of a *sae2* mutant is not as strong as for *sin3* (Figure 3D). Other factors, currently unknown, are also proposed to be regulated by Rpd3 and Hda1 and to contribute to expansions by mechanisms that remain to be elucidated (Figure 3E). Sae2 and Mre11 (acting in the Mre11/Rad50/Xrs2 complex) are known to process hairpin DNA in vivo and in vitro [33],[34]. It remains to be determined whether these enzymes actually process a TNR hairpin intermediate to accelerate expansions. The effects of Sae2 and Mre11 have also been examined for expansions of long (CAG)_70_ repeats [19]. In this study, expansion frequencies increased in *sae2* or *mre11* mutants. One likely explanation is that long alleles in yeast break more frequently than do the shorter alleles we utilize; thus, long repeats in yeast rely on double strand break repair to prevent expansions [19]. In support of this possibility, expansions of (CAG)_70_ are also enhanced by loss of the recombination proteins Rad51 and Rad52 [19], whereas *rad51* or *rad52* mutants do not affect expansion rates of CTG alleles between 13 and 25 repeats [35],[36]. The outcomes of Sae2 and Mre11 activity could be different in break repair than in putative hairpin processing described above.

We found that yeast HDAC mutants suppress expansions in nearly all assays (Figure 1C), but quantitative differences in phenotype illustrate that some aspects of HDAC regulation of expansions remain unknown. What other factors regulated by yeast Rpd3L and Hda1 or human HDAC3 might contribute to expansions? One possibility is chromatin structure near but not immediately adjacent to the repeat. The triplet repeat literature contains several connections between expansions and proteins that modulate chromatin structure, including *Drosophila* CBP [22] mentioned above, the insulator protein CTCF [37],[38], and the DNA methyltransferase Dnmt1 [39]. A second possibility is that HDACs promote expansions by controlling the firing of DNA replication origins [40]–[43]. The major finding against the origin firing model is that similar *SIN3*-dependent promotion of expansions was seen when our yeast reporter was integrated at four different loci (*LYS2*, *INO1*, *SPS2*, and *IME2*; Figures 1 and [3]), which are 21–130 kb away from the nearest origins that become deregulated in *rpd3Δ* cells [42]. We feel it is unlikely that Rpd3-dependent origin firing explains suppression of expansions, although HDAC effects on fork progression or fork stalling cannot be ruled out at this time.

HDAC inhibitors are currently being evaluated as therapies to treat the transcriptional defects in several TNR expansion diseases [44],[45]. For example, **4b** treatment reverses *FXN* gene silencing in primary cells from Friedreich's ataxia patients [24] and relieves disease phenotype and transcriptional abnormalities in HD transgenic mice [25]. Our work implies these inhibitors may have a second, beneficial effect of suppressing somatic expansions that contribute to disease progression.

## Materials and Methods

### Genetic Assays and Analysis of Expanded TNR Alleles

Triplet repeat expansion assays using the *URA3* reporter have been described previously [18],[27]. Assays using the *CAN1* reporter (Figure 1A) utilized canavanine at 60 µg/ml to select for resistance. All expansions were verified by single-colony PCR across the repeat tract followed by analysis on high-resolution polyacrylamide gels [18]. Details of statistical analysis are provided in Tables S1 and S4.

### Western Blot Analysis

Whole cell lysates (yeast and SVG-A astrocytes) or histone acid extracts (SVG-A astrocytes) were separated electrophoretically and transferred to PVDF membranes. Primary rabbit antibodies were against histone H3 (A300-823A, Bethyl Laboratories), acetyl-histone H3 (#17-615, Millipore), acetyl-histone H4 (#06-866, Millipore), β-actin (A2066, Sigma-Aldrich), HDAC3 (sc-11417, Santa Cruz Biotechnology), and HDAC1 (CH00218, Coriell Institute for Medical Research). Assessment of HDAC3 expression via Western blot analysis resulted in two bands around 50 kDa, the predicted size of the protein, presumably representing the two reported isoforms of HDAC3 [46]. Throughout all experiments, consistent knockdown of the top band was observed following HDAC3 siRNA treatment, however levels of the bottom band varied between experiments. Quantitation of HDAC3 knockdown was performed by densitometric analysis of the top band only. A mouse antibody was used against histone H4 (ab31830, Abcam). Secondary antibodies conjugated to horseradish peroxidase were 711-035-152 and 115-035-003 from Jackson ImmunoResearch Laboratories. Visualization was by chemilluminescence (Western Lightning Plus-ECL, PerkinElmer).

### Chromatin Immunoprecipitation

250 ml yeast cell cultures were grown to A_600_∼0.8 at 30° in yeast extract/peptone/dextrose. Following cross-linking with 1% formaldehyde (15 min, 22°), cross-linked chromatin was isolated in lysis buffer containing 50 mM HEPES/KOH pH 7.5, 140 mM NaCl, 1 mM EDTA, 1% Triton X-100%, 0.1% sodium deoxycholate and the protease inhibitors 1 mM PMSF, 1 mM benzamidine, 1 µg/ml leupeptin, and 1 µg/ml pepstatin. After sonication (40% duty cycle for seven cycles of 5 s each with 50 s cooling in between; Digital Sonifier EDP 100-214-239, Branson), chromatin fragments were immunoprecipitated with antibodies specific for total histone H4 (5 µg, A300-646A, Bethyl Laboratories) or pan-acetylated H4 (7 µl, # 06-866, Millipore) at 4°C overnight. Immune complexes were captured by incubating with Protein G magnetic beads (S1430S, New England BioLabs) for 4 h at 4°C. After a series of washes, DNA was eluted in 250 µl elution buffer (50 mM Tris-HCl pH 8, 10 mM EDTA, and 1% SDS) and crosslinks were reversed by incubating overnight at 65°C. DNA was purified by phenol-chloroform extraction followed by an ethanol precipitation and analyzed by quantitative PCR (Applied Biosystems, 7500 FAST). Primer sequences used for quantitative PCR are provided in the Supporting Information section. Signals for total H4 and acetylated H4 were quantified by the method of 2^−ΔΔCt^ and normalized using the following calculation: (C_t_ immunoprecipitate−C_t_ input)−(C_t_ background−C_t_ input). Amplification of the chromosome VI telomere region was chosen as a measurement for background [31],[47]. The normalized IP values obtained for acetylated H4 were divided by the normalized IP values for total H4.

### Reverse Transcription-PCR

Cells were grown to mid-log phase and then extracted with hot acidic phenol. Following clean-up of the RNA, reverse transcription was performed in triplicate. cDNA levels were analyzed in triplicate by quantitative real-time PCR and normalized to *ALG9* levels. Details and primer sequences are provided in Table S2.

### Shuttle Vector Assays and Molecular Analysis of Protein Components

SVG-A astrocytes were seeded in 60 mm tissue culture dishes and transfected with 5 µg shuttle vector DNA using Lipofectamine 2000 (Invitrogen Corporation). After 6 h, the DMEM transfection media was replaced by DMEM supplement with 10% fetal bovine serum, plus one of the HDAC inhibitors **4b** or compound **3** (kindly provided by Joel Gottesfeld, The Scripps Research Institute) or DMSO only. Cells were incubated for an additional 48 hours, then samples were taken for either expansion assay or histone analysis. To measure expansions, plasmid DNA was extracted and concentrated by using Hirt's alkaline lysis [48] and Amicon Ultra 50 K centrifugal filter units (Millipore). Purified plasmid DNA was digested by DpnI (New England Biolabs) and then transformed into *S. cerevisiae* for measurement of canavanine resistance or into *E. coli* for analysis of total plasmid numbers as measured by ampicillin-resistant colonies. Histone extracts were prepared by acid extraction (protocol provided by Abcam).

RNA interference experiments were performed with minor variations. SVG-A cells were seeded and transfected with ON-TARGET plus or siGenome SMARTpool siRNAs (100 nM) against HDAC3 (L-003496, M-003496), HDAC1 (M-003493), or scrambled non-targeting siRNA (D-001810) from Dharmacon using DharmaFECT 1. After 48 h, cells were transfected with 7 µg of shuttle vector and also re-transfected with siRNAs using Lipofectamine 2000. After another 2 d, expansion frequencies were prepared as above, in parallel with immunoblot analysis of whole cell lysates.

### Statistical Analyses

All *p* values were determined by two-tailed Student's *t* test. *p* and *n* values for each data set are specified in Tables S1, S2, S3, S4 unless stated in the figure legend.

## Supporting Information

Figure S1 Identification of mutants with reduced expansion rates. (A) Overview of screen and results. (B) Schematic of replica plating strategy to identify relevant mutants.
(TIF)

Figure S2 Expansion sizes in yeast ± TSA. Expansion sizes were measured by PCR and high-resolution gel electrophoresis to within ±2 repeats. All expansions are genetically independent. The histogram shows the spectra from 42 expansions seen in cells treated with DMSO (unfilled bars), or from 39 expansions from cells treated with 30 µg/ml TSA (blue-filled bars).
(TIF)

Figure S3 Survival of *sin3*, *pho23*, and *hda3* mutants on canavanine- or 5FOA-containing media. This experiment tests whether HDAC mutants without a triplet repeat reporter show any innate sensitivity to canavanine or 5FOA, the compounds used to select expansions from the *CAN1* and *URA3* reporters, respectively. If there were any innate sensitivity, then expansion assays with the HDAC mutants might give low apparent expansion rates for reasons unrelated to the triplet repeats themselves. For each strain, spontaneous deletion of the reporter (“pop-out”) was identified genetically. Cells from each reporter-less strain were grown in YPD medium to mid-log phase, and serial 10-fold dilutions were spotted onto control media (SC-Ura, left) or selective media (center and right). The plates were incubated at 30° for 6 d and then photographed. Selection was for canavanine resistance (top) or 5FOA resistance (bottom). Low concentrations of Can or 5FOA were used to magnify any difference in sensitivities of wild type controls versus HDAC mutants. The results indicate similar growth rates for wild type and HDAC mutants on the control media (left) and plates with low (center) or high drug concentrations (center). Based on these experiments, we conclude there is no evidence for innate sensitivity of the HDAC mutants to canavanine or 5FOA. Therefore, low expansion rates in the HDAC mutants cannot be attributed to the selection method.
(TIF)

Figure S4 Growth tests of *sin3*, *pho23*, and *hda3* mutants containing an expanded repeat on canavanine-containing media. This experiment tests whether HDAC mutants with an expanded CTG repeat grow similarly to wild type on selective media. The result will tell whether a hypothetical slow-growth phenotype in HDAC mutants on selective media could lead to undercounting of Can resistant colonies, thus imitating low expansion rates. For each strain, a spontaneous expansion was identified that contained circa 33 CTG repeats, based on PCR analysis (Figure 1B). The cells were then resuspended in water, and serial 10-fold dilutions were spotted onto complete media (top panel) or canavanine-containing media. The cells were incubated at 30° for 2 d (top panel) or 6 d (bottom panel). The time, temperature, and selective media are all the same as used when measuring expansion rates. The results indicate similar growth rates, and clearly visible colonies, for all the HDAC mutants and the wild type control strain. We conclude that the reduced expansion rates in the HDAC mutants cannot be attributed to slow growth on canavanine-containing media.
(TIF)

Figure S5 Expansion rate data for alternative HDACs. This experiment tests whether mutation of any HDAC besides Rpd3L or Hda1 gives reduced rates of expansion for the (CTG)20-*CAN1* reporter integrated on chromosome II. For each strain, expansion rates were measured as described in Materials and Methods. Data for *sin3*, *pho23*, and *hda3* strains are reproduced from Figure 1C for comparison. Error bars represent ±1 SEM. The results indicate that the additional HDAC mutants tested yielded small expansion phenotypes compared to *sin3*, *pho23*, or *hda3*.
(TIF)

Figure S6 Accumulation of acetylated histone H4 upon treatment of SVG-A cells with the HDAC inhibitor 4b. These results are from four independent measurements of acetylated histone H4 (AcH4) and total H4 by immunoblot. One representative blot is shown in Figure 2E. The graph below shows the AcH4/Total H4 ratio normalized to the DMSO-only control. Error bars denote ±1 SEM. * *p*<0.05 compared to untreated.
(TIF)

Figure S7 Histone acetylation levels at *LYS2*. Chromatin immunoprecipitation (ChIP) was used to measure acetylated histone H4 (AcH4) and total H4 levels. Results were measured by real-time PCR of the *LYS2* promoter. Primer positions for each gene are shown in Figure S8. The *x*-axis indicates strains with the TNR reporter integrated at different genomic loci. Bars are average of three measurements. Error bars reflect ±1 SEM.
(TIF)

Figure S8 Position of ChIP primers. Real-time PCR was used to quantify the ChIP signals in Figure 3 and Figure S7. Shown below are the primer positions (not to scale) when the TNR reporter was integrated at the query loci. The 4.3–6 kb distance between the query site primers and the TNR primers make it likely that the two amplicons were derived from independent template fragments. In each case the target locus was disrupted by the reporter; for example *IN…O1* indicates disruption of the *INO1* gene.
(TIF)

Table S1 Expansion rate analysis in yeast HDAC mutants. All rate data are expressed as expansions per cell generation. *n*, number of independent rate measurements; SEM, standard error of the mean; *p* values calculated by Student's *t* test.
(TIF)

Table S2 Expansion suppression and transcript levels in HDAC mutants. Expansion suppression values are from Table S1. Transcript levels were measured in triplicate from three independent cDNA preparations. For RNA preparation, yeast cells from overnight cultures were grown in YPD to an A600 of 0.6. Cultures were then centrifuged at room temperature for 5 min at 4,000 rpm, washed in sterile water, and centrifuged again. RNA extraction was performed using hot acidic phenol as described previously (http://www.transcriptome.ens.fr/sgdb/protocols/preparation_yeast.php). A maximum of 100 µg of RNA was used for clean-up. The RNeasy Mini Kit (Qiagen) was used for the RNA clean up, which included the on-column DNase digestion. 1 µg of total RNA was reverse-transcribed in triplicate into cDNA using random nonamer primers in a 20 µl reaction mixture using the Primerdesign Precision qScript Reverse Transcription kit. The cDNA levels were then analyzed using the Applied Biosystems 7500 FAST. Each cDNA sample replicate was tested in triplicate in a 96-well plate, and values were normalized to *ALG9* expression. The reaction mix consisted of 10 µl of Fast SYBR Green Master Mix (Applied Biosystems) in final volume of 20 µl. A blank (no template control) was also incorporated into each assay. Relative expression levels were determined using the method of 2^−ΔΔCt^. Primer sequences were: rtCAN1F, CGA ATG GCT ATT AAA TAT CAC TGG TGT TGC; rtCAN1R, GAA TTT TGG TGC AAA AGC CGT GAA ACC TTG; rtALG9F, CAC GGA TAG TGG CTT TGG TGA ACA ATT AC; rtALG9R, TAT GAT TAT CTG GCA GCA GGA AAG AAC TTG GG.
(TIF)

Table S3 Expansion frequencies in SVG-A cells. Expansion frequencies (defined in Materials and Methods and in the legend to Figure 2) were normalized to frequencies from control cells, as indicated. *n*, number of independent experiments; SEM, standard error of the mean. *p* values were calculated by two-tailed Student's *t* test. Background expansions were estimated at 0.06±0.04 relative expansion frequency. Absolute frequencies of expansions, expressed as verified expansions per 100,000 *E. coli* transformants, were: 44±22 for HDAC inhibitor 4b experiments; 220±24 for HDAC3 and HDAC1 knockdown experiments; and 120±76 for CBP, p300, and CBP+p300 knockdowns. Knockdown efficiencies were estimated by immunoblot at 75%–80% for single knockdowns of CBP or p300, and 80%–85% each for the double knockdown.
(TIF)

Table S4 Data for expansions and ChIP at *INO1* and *SPS2*, and expansion rate analysis in yeast nuclease mutants. *n*, number of independent experiments; SEM, standard error of the mean; *p* values calculated by two-tailed Student's *t* test.
(TIF)

## Abbreviations

DM1: myotonic dystrophy type 1
HAT: histone acetyltransferase
HD: Huntington′s disease
HDAC: histone deacetylase complex
TNR: trinucleotide repeat
TSA: trichostatin A
