# Supplementary figures and images for: Histone Deacetylase Complexes Promote Trinucleotide Repeat Expansions
Source: PLoS Biol. 2012 Feb 21;10(2):e1001257. doi: 10.1371/journal.pbio.1001257 (PMC3283555; doi:10.1371/journal.pbio.1001257)

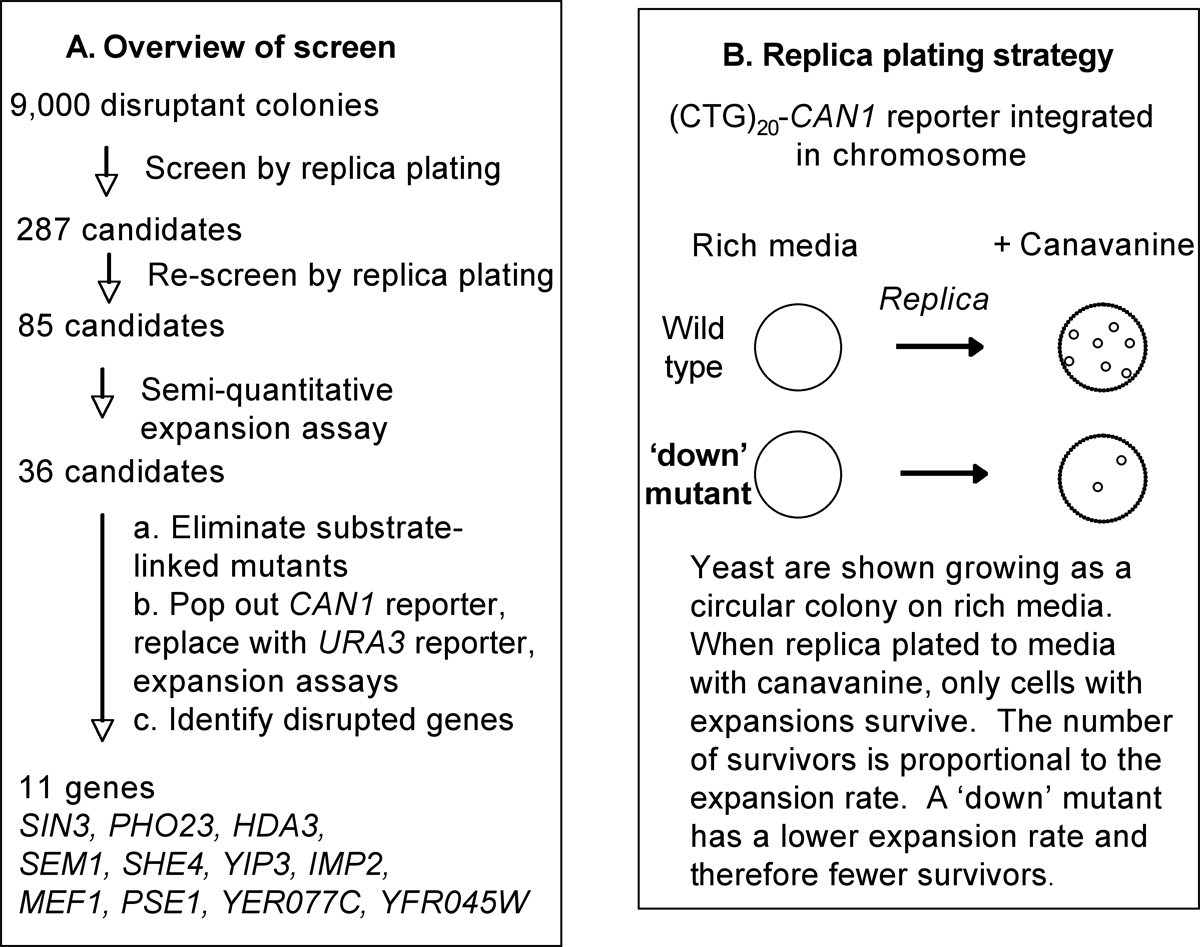

Supplement: Figure S1 — Identification of mutants with reduced expansion rates. (A) Overview of screen and results. (B) Schematic of replica plating strategy to identify relevant mutants. (TIF) [file pbio.1001257.s001.tif]

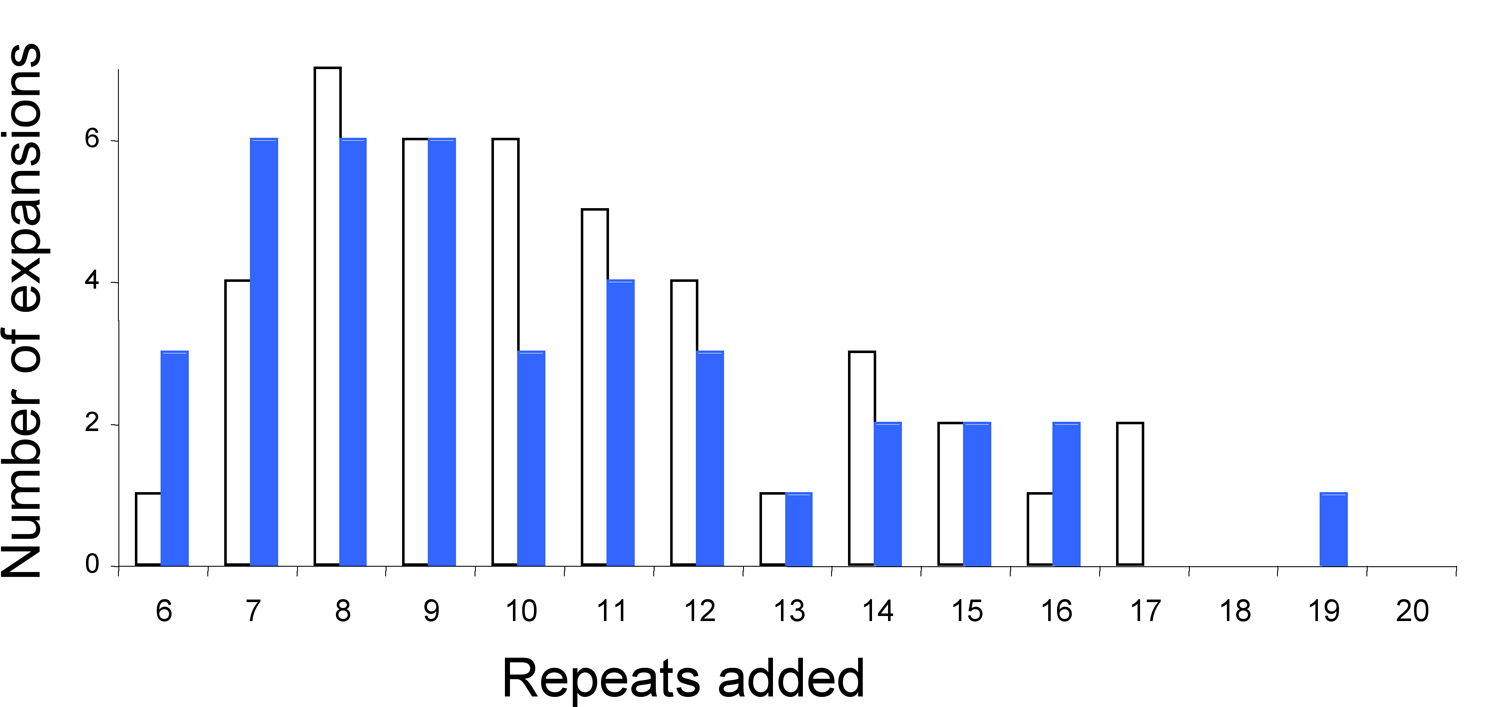

Supplement: Figure S2 — Expansion sizes in yeast ± TSA. Expansion sizes were measured by PCR and high-resolution gel electrophoresis to within ±2 repeats. All expansions are genetically independent. The histogram shows the spectra from 42 expansions seen in cells treated with DMSO (unfilled bars), or from 39 expansions from cells treated with 30 µg/ml TSA (blue-filled bars). (TIF) [file pbio.1001257.s002.tif]

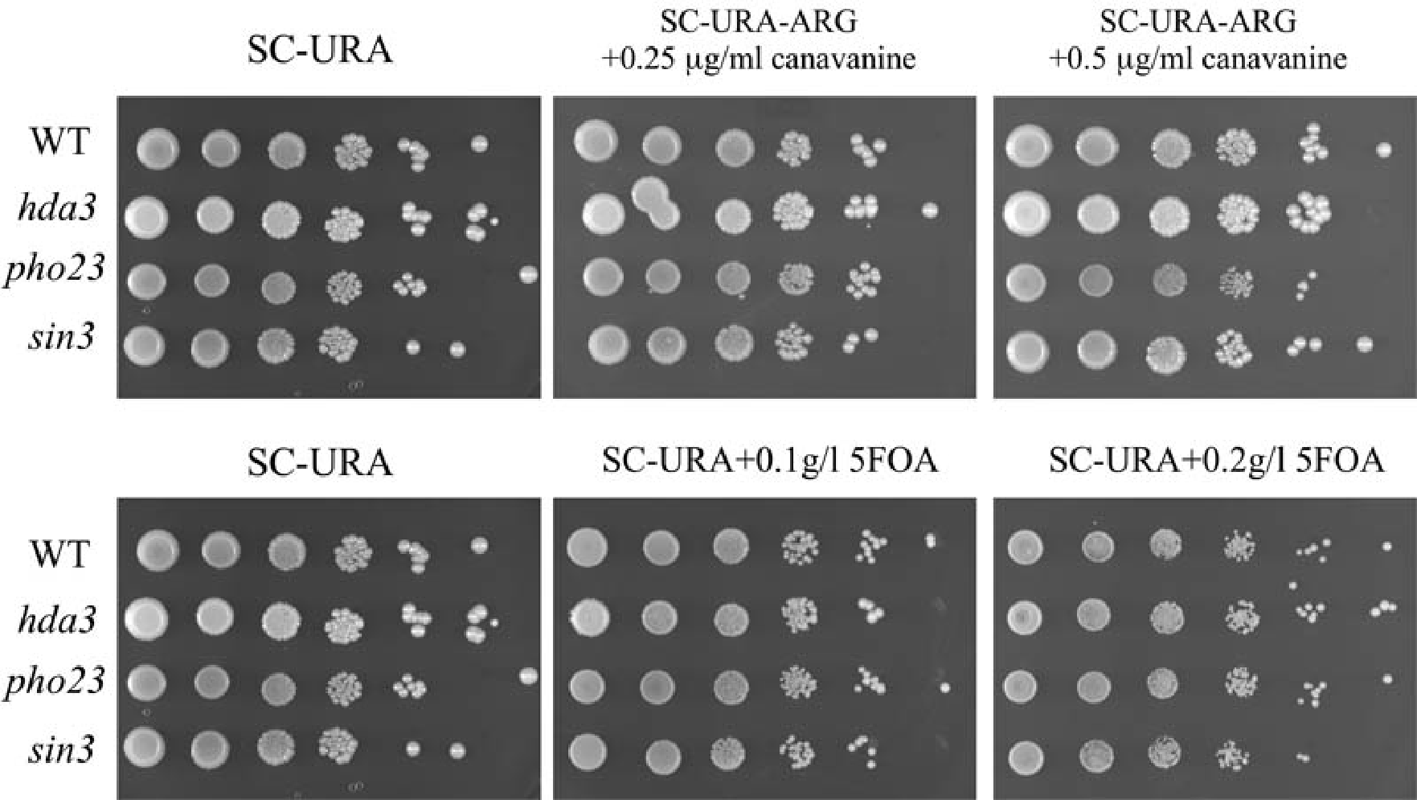

Supplement: Figure S3 — Survival of sin3, pho23, and hda3 mutants on canavanine- or 5FOA-containing media. This experiment tests whether HDAC mutants without a triplet repeat reporter show any innate sensitivity to canavanine or 5FOA, the compounds used to select expansions from the CAN1 and URA3 reporters, respectively. If there were any innate sensitivity, then expansion assays with the HDAC mutants might give low apparent expansion rates for reasons unrelated to the triplet repeats themselves. For each strain, spontaneous deletion of the reporter (“pop-out”) was identified genetically. Cells from each reporter-less strain were grown in YPD medium to mid-log phase, and serial 10-fold dilutions were spotted onto control media (SC-Ura, left) or selective media (center and right). The plates were incubated at 30° for 6 d and then photographed. Selection was for canavanine resistance (top) or 5FOA resistance (bottom). Low concentrations of Can or 5FOA were used to magnify any difference in sensitivities of wild type controls versus HDAC mutants. The results indicate similar growth rates for wild type and HDAC mutants on the control media (left) and plates with low (center) or high drug concentrations (center). Based on these experiments, we conclude there is no evidence for innate sensitivity of the HDAC mutants to canavanine or 5FOA. Therefore, low expansion rates in the HDAC mutants cannot be attributed to the selection method. (TIF) [file pbio.1001257.s003.tif]

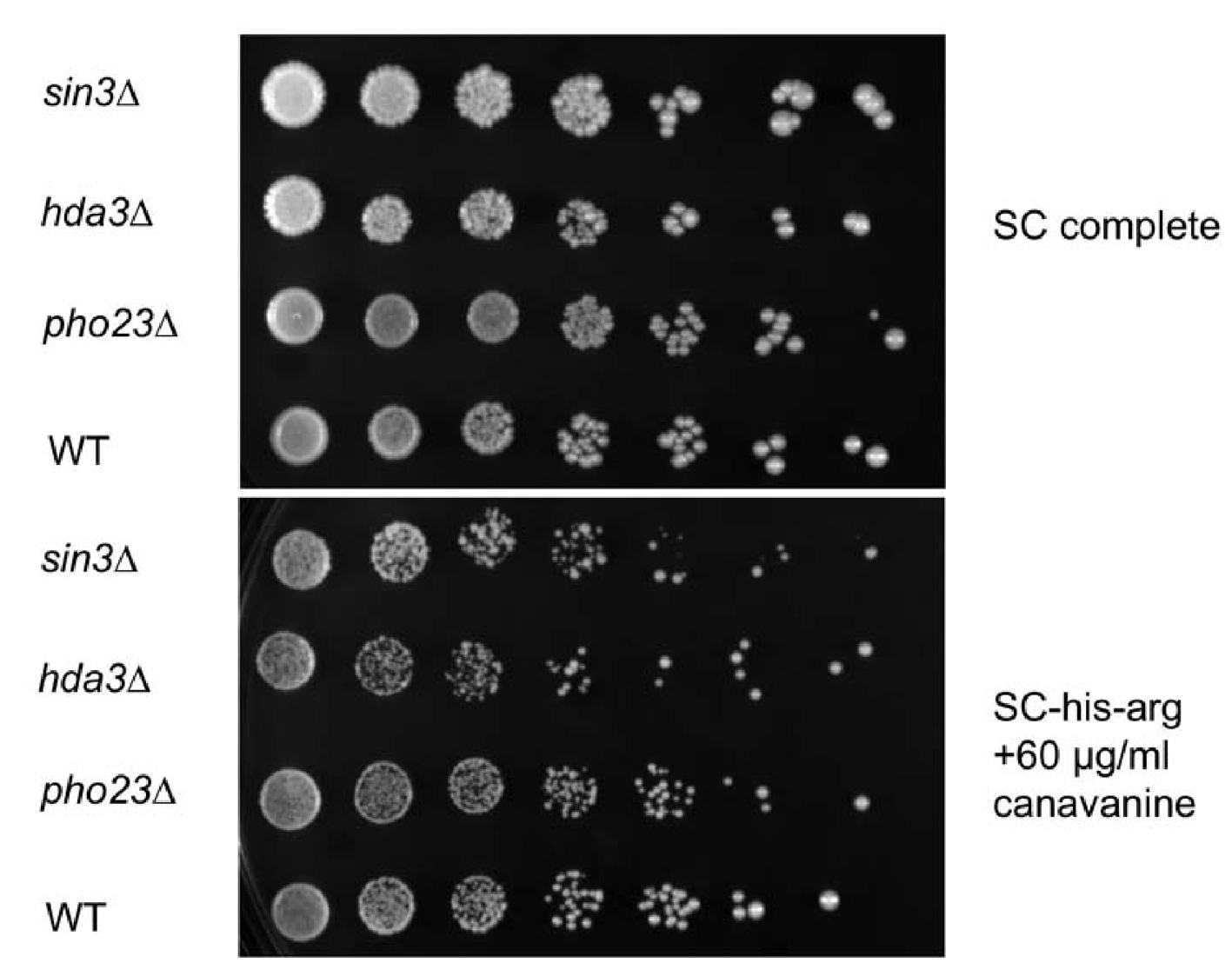

Supplement: Figure S4 — Growth tests of sin3, pho23, and hda3 mutants containing an expanded repeat on canavanine-containing media. This experiment tests whether HDAC mutants with an expanded CTG repeat grow similarly to wild type on selective media. The result will tell whether a hypothetical slow-growth phenotype in HDAC mutants on selective media could lead to undercounting of Can resistant colonies, thus imitating low expansion rates. For each strain, a spontaneous expansion was identified that contained circa 33 CTG repeats, based on PCR analysis (Figure 1B). The cells were then resuspended in water, and serial 10-fold dilutions were spotted onto complete media (top panel) or canavanine-containing media. The cells were incubated at 30° for 2 d (top panel) or 6 d (bottom panel). The time, temperature, and selective media are all the same as used when measuring expansion rates. The results indicate similar growth rates, and clearly visible colonies, for all the HDAC mutants and the wild type control strain. We conclude that the reduced expansion rates in the HDAC mutants cannot be attributed to slow growth on canavanine-containing media. (TIF) [file pbio.1001257.s004.tif]

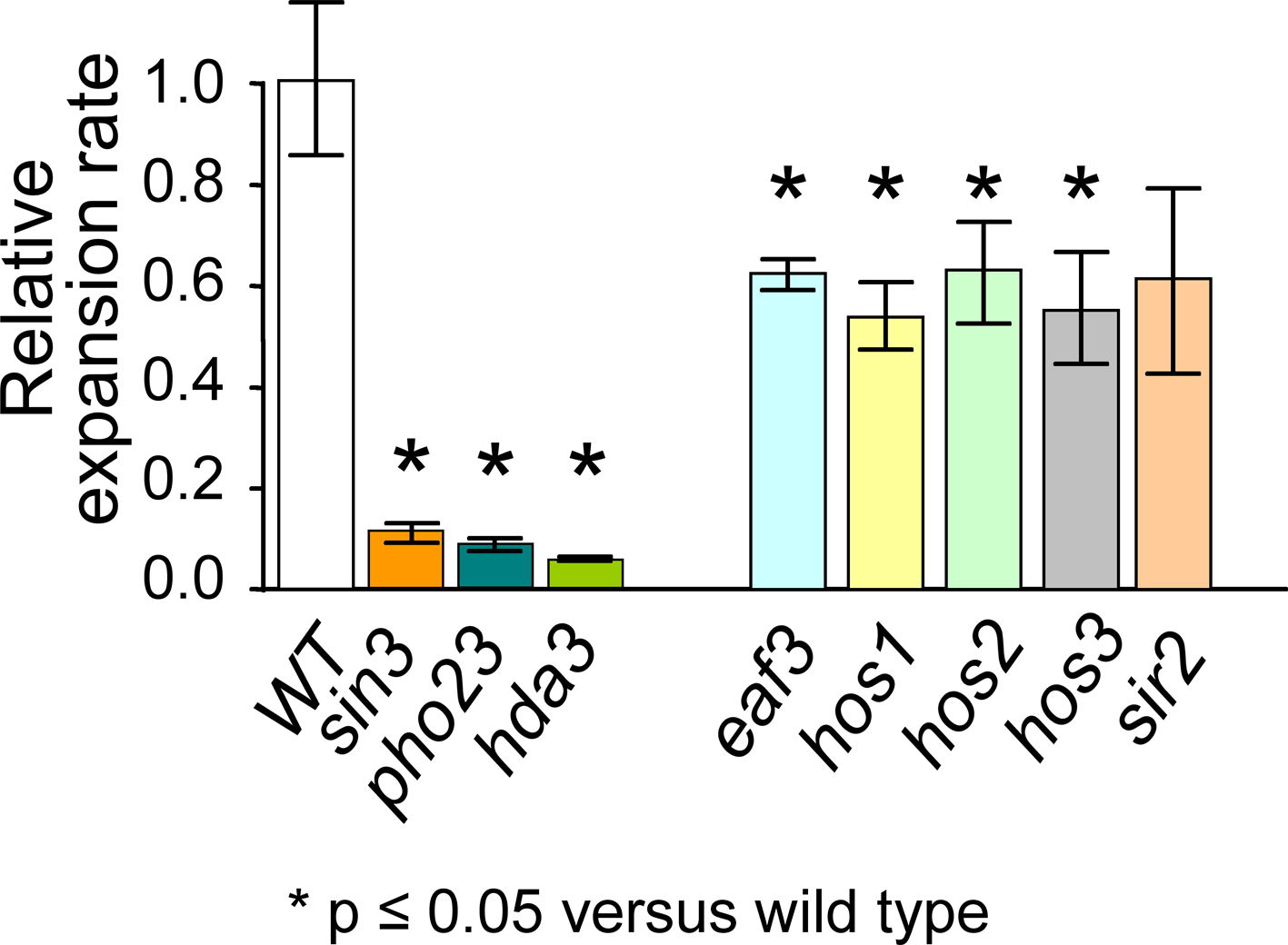

Supplement: Figure S5 — Expansion rate data for alternative HDACs. This experiment tests whether mutation of any HDAC besides Rpd3L or Hda1 gives reduced rates of expansion for the (CTG)20-CAN1 reporter integrated on chromosome II. For each strain, expansion rates were measured as described in Materials and Methods. Data for sin3, pho23, and hda3 strains are reproduced from Figure 1C for comparison. Error bars represent ±1 SEM. The results indicate that the additional HDAC mutants tested yielded small expansion phenotypes compared to sin3, pho23, or hda3. (TIF) [file pbio.1001257.s005.tif]

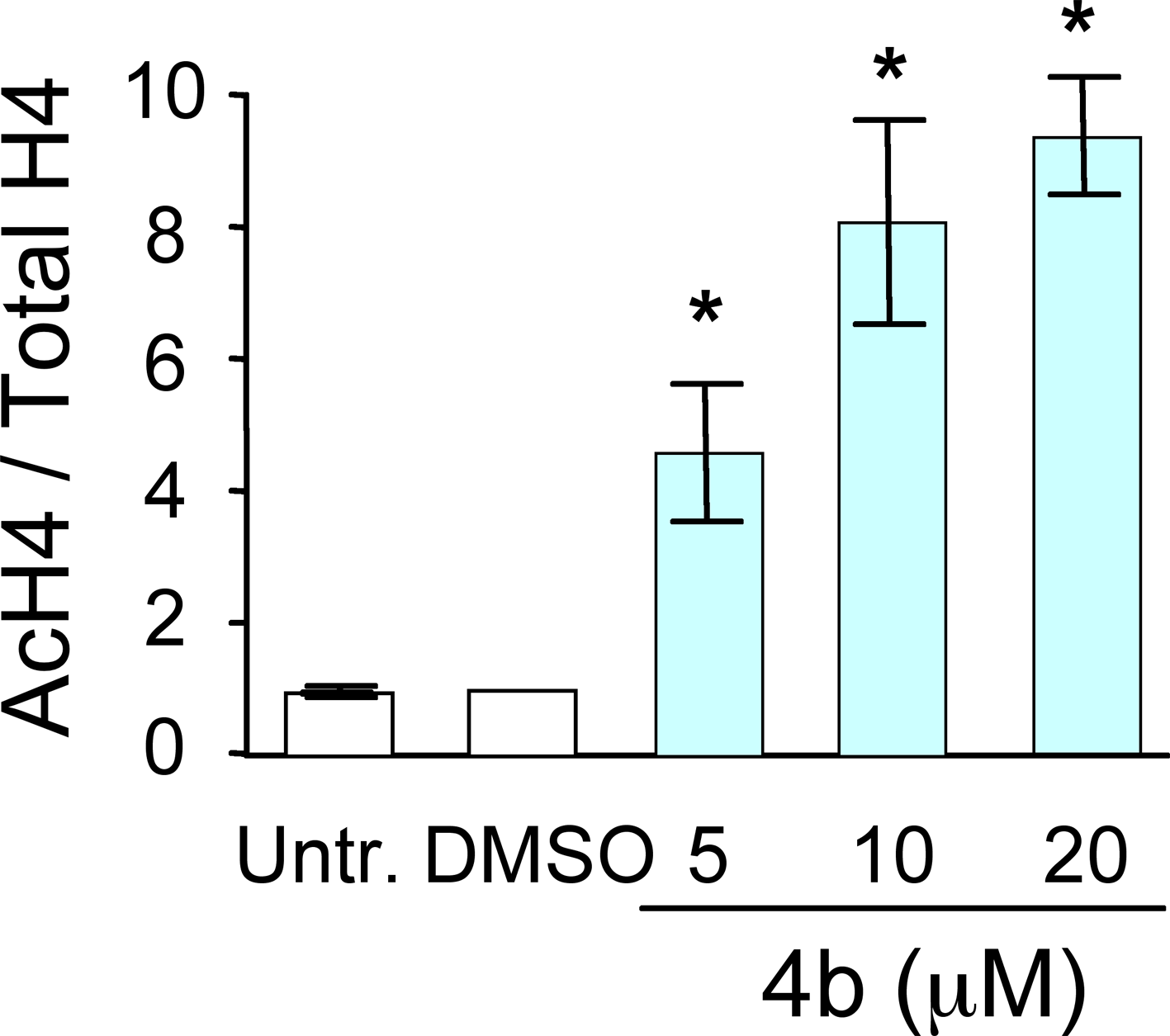

Supplement: Figure S6 — Accumulation of acetylated histone H4 upon treatment of SVG-A cells with the HDAC inhibitor 4b. These results are from four independent measurements of acetylated histone H4 (AcH4) and total H4 by immunoblot. One representative blot is shown in Figure 2E. The graph below shows the AcH4/Total H4 ratio normalized to the DMSO-only control. Error bars denote ±1 SEM. * p<0.05 compared to untreated. (TIF) [file pbio.1001257.s006.tif]

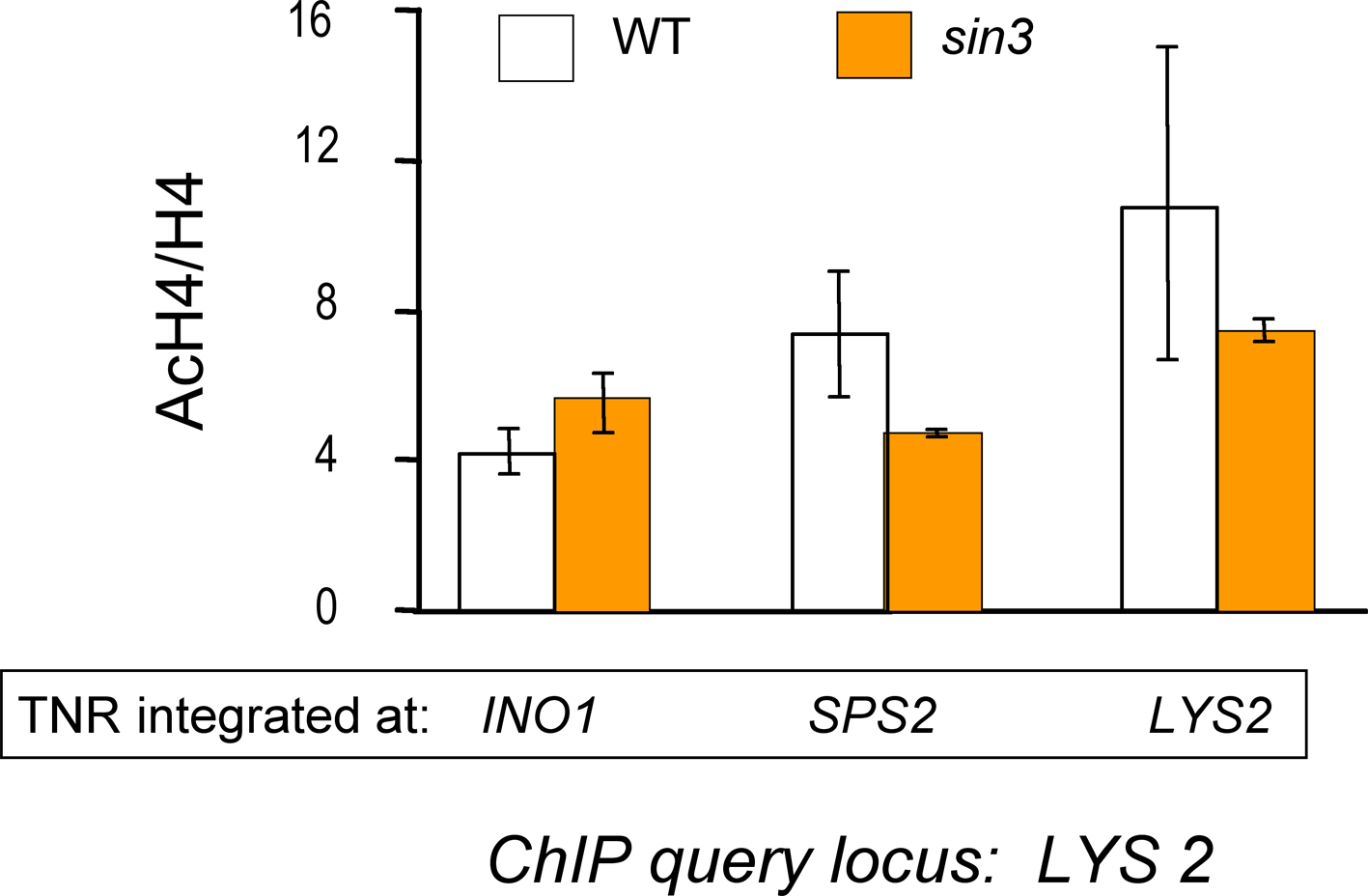

Supplement: Figure S7 — Histone acetylation levels at LYS2. Chromatin immunoprecipitation (ChIP) was used to measure acetylated histone H4 (AcH4) and total H4 levels. Results were measured by real-time PCR of the LYS2 promoter. Primer positions for each gene are shown in Figure S8. The x-axis indicates strains with the TNR reporter integrated at different genomic loci. Bars are average of three measurements. Error bars reflect ±1 SEM. (TIF) [file pbio.1001257.s007.tif]

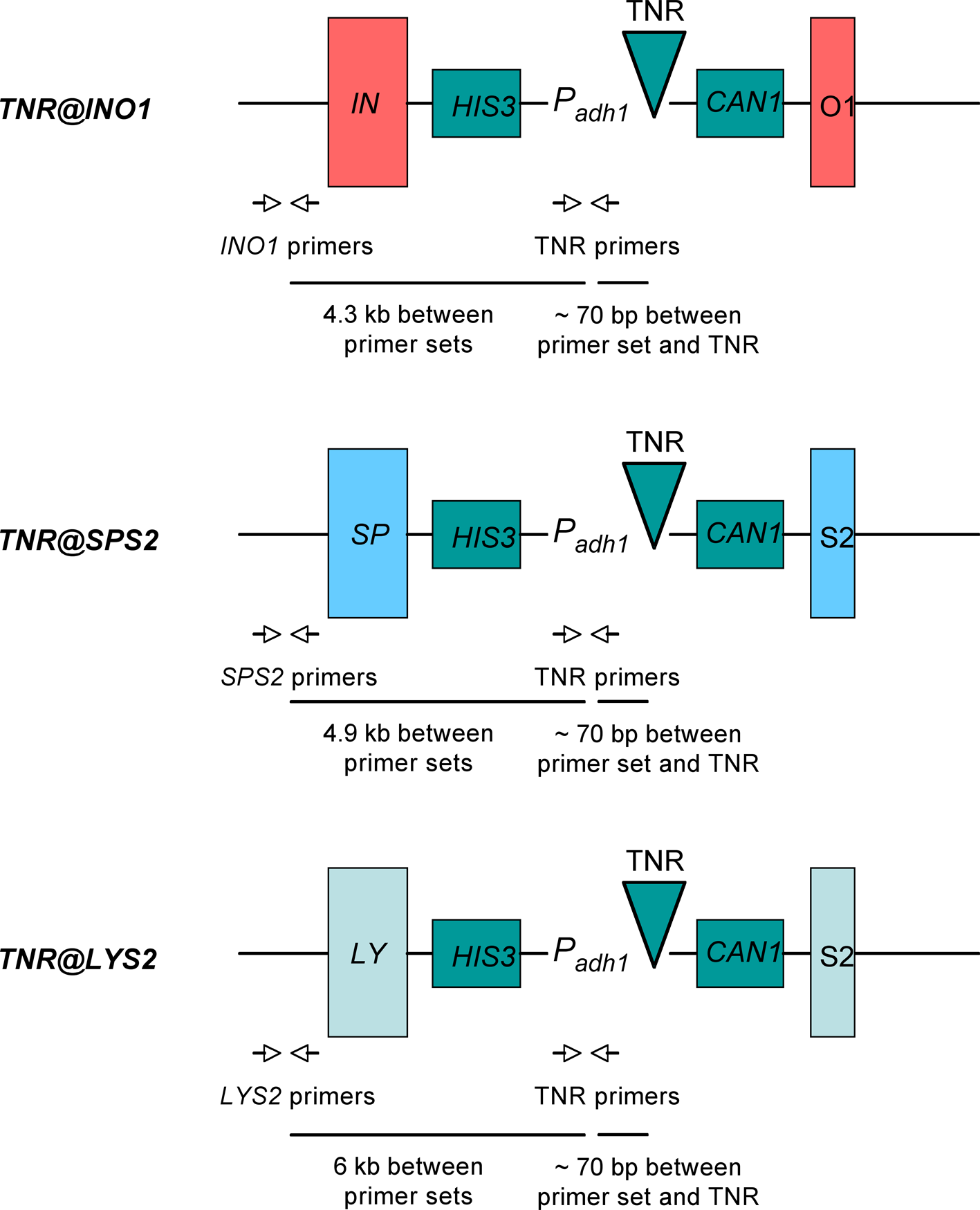

Supplement: Figure S8 — Position of ChIP primers. Real-time PCR was used to quantify the ChIP signals in Figure 3 and Figure S7. Shown below are the primer positions (not to scale) when the TNR reporter was integrated at the query loci. The 4.3–6 kb distance between the query site primers and the TNR primers make it likely that the two amplicons were derived from independent template fragments. In each case the target locus was disrupted by the reporter; for example IN…O1 indicates disruption of the INO1 gene. (TIF) [file pbio.1001257.s008.tif]

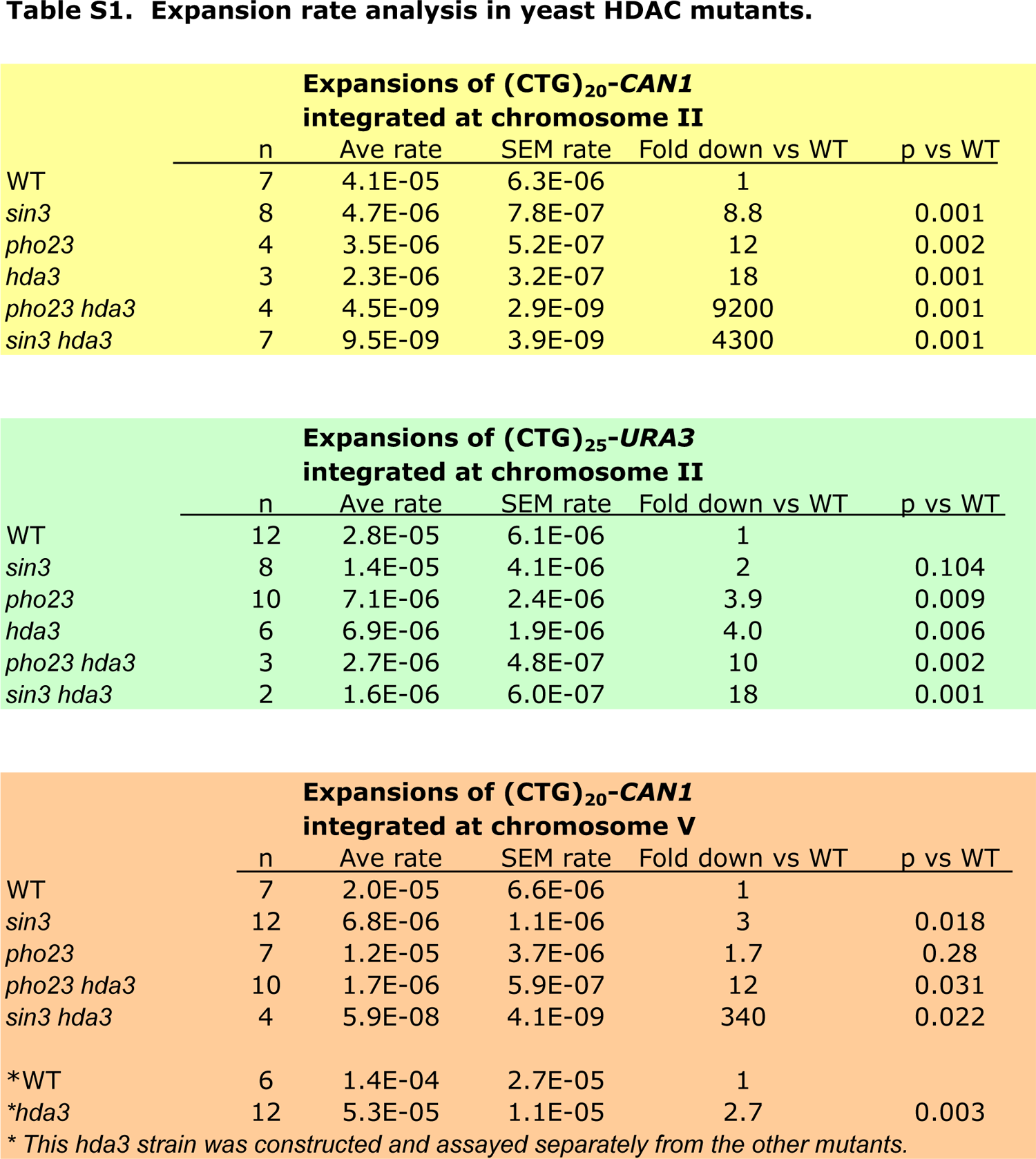

Supplement: Table S1 — Expansion rate analysis in yeast HDAC mutants. All rate data are expressed as expansions per cell generation. n, number of independent rate measurements; SEM, standard error of the mean; p values calculated by Student's t test. (TIF) [file pbio.1001257.s009.tif]

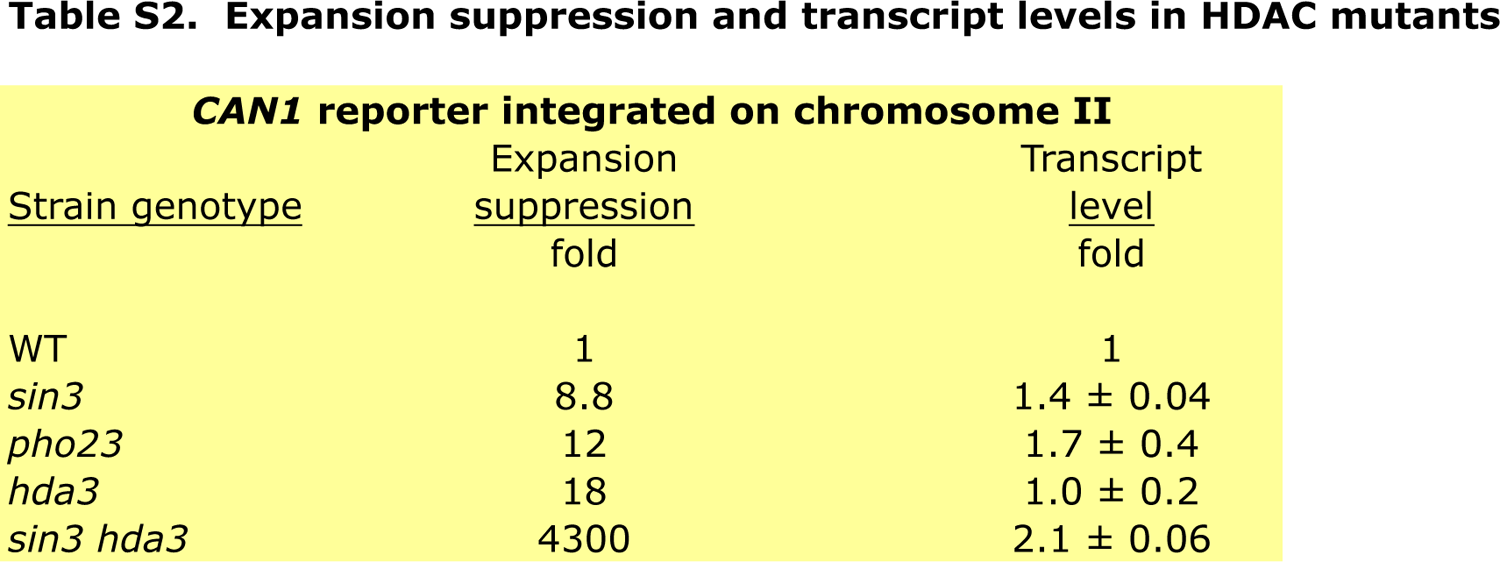

Supplement: Table S2 — Expansion suppression and transcript levels in HDAC mutants. Expansion suppression values are from Table S1. Transcript levels were measured in triplicate from three independent cDNA preparations. For RNA preparation, yeast cells from overnight cultures were grown in YPD to an A600 of 0.6. Cultures were then centrifuged at room temperature for 5 min at 4,000 rpm, washed in sterile water, and centrifuged again. RNA extraction was performed using hot acidic phenol as described previously (http://www.transcriptome.ens.fr/sgdb/protocols/preparation_yeast.php). A maximum of 100 µg of RNA was used for clean-up. The RNeasy Mini Kit (Qiagen) was used for the RNA clean up, which included the on-column DNase digestion. 1 µg of total RNA was reverse-transcribed in triplicate into cDNA using random nonamer primers in a 20 µl reaction mixture using the Primerdesign Precision qScript Reverse Transcription kit. The cDNA levels were then analyzed using the Applied Biosystems 7500 FAST. Each cDNA sample replicate was tested in triplicate in a 96-well plate, and values were normalized to ALG9 expression. The reaction mix consisted of 10 µl of Fast SYBR Green Master Mix (Applied Biosystems) in final volume of 20 µl. A blank (no template control) was also incorporated into each assay. Relative expression levels were determined using the method of 2−ΔΔCt. Primer sequences were: rtCAN1F, CGA ATG GCT ATT AAA TAT CAC TGG TGT TGC; rtCAN1R, GAA TTT TGG TGC AAA AGC CGT GAA ACC TTG; rtALG9F, CAC GGA TAG TGG CTT TGG TGA ACA ATT AC; rtALG9R, TAT GAT TAT CTG GCA GCA GGA AAG AAC TTG GG. (TIF) [file pbio.1001257.s010.tif]

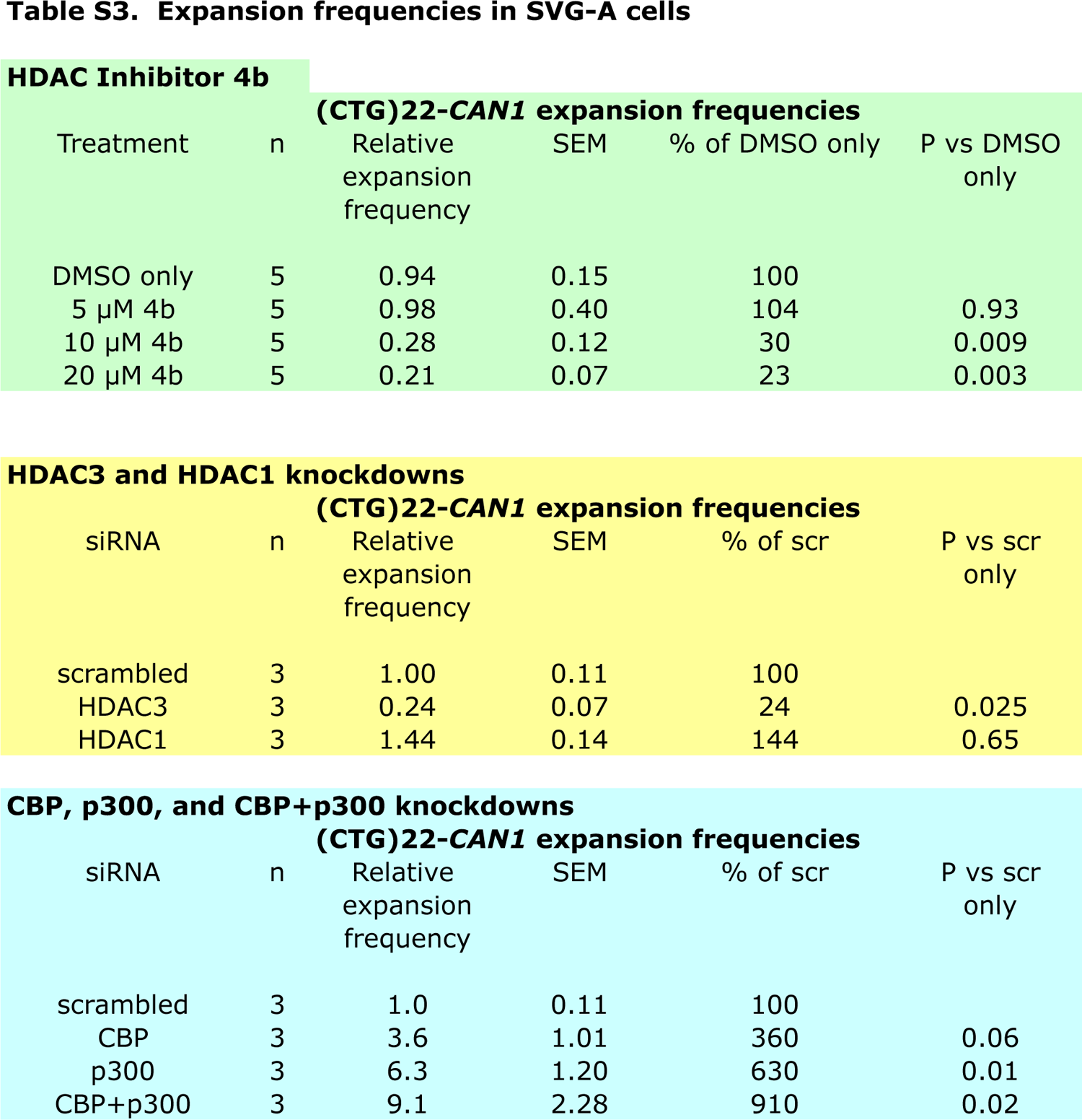

Supplement: Table S3 — Expansion frequencies in SVG-A cells. Expansion frequencies (defined in Materials and Methods and in the legend to Figure 2) were normalized to frequencies from control cells, as indicated. n, number of independent experiments; SEM, standard error of the mean. p values were calculated by two-tailed Student's t test. Background expansions were estimated at 0.06±0.04 relative expansion frequency. Absolute frequencies of expansions, expressed as verified expansions per 100,000 E. coli transformants, were: 44±22 for HDAC inhibitor 4b experiments; 220±24 for HDAC3 and HDAC1 knockdown experiments; and 120±76 for CBP, p300, and CBP+p300 knockdowns. Knockdown efficiencies were estimated by immunoblot at 75%–80% for single knockdowns of CBP or p300, and 80%–85% each for the double knockdown. (TIF) [file pbio.1001257.s011.tif]

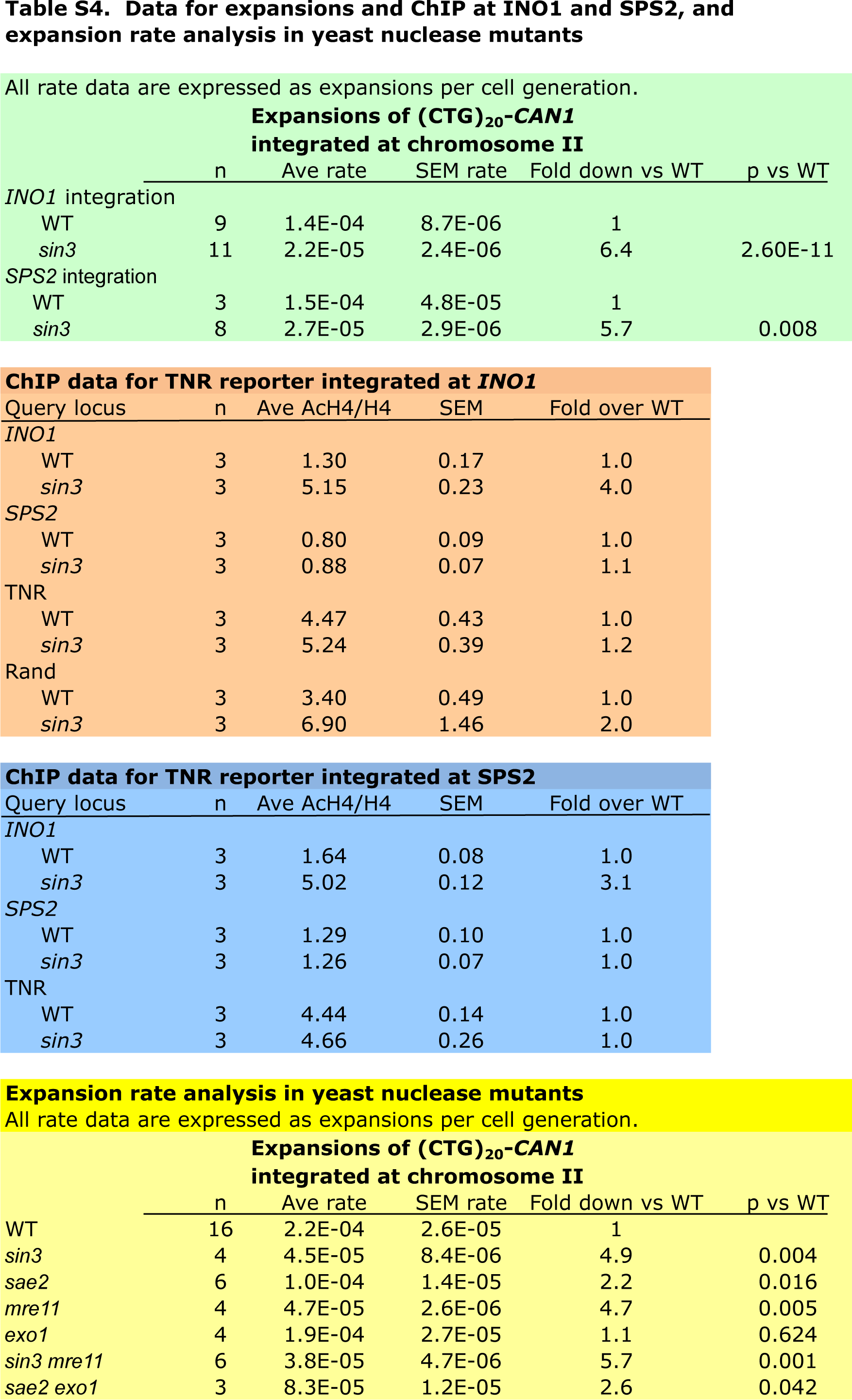

Supplement: Table S4 — Data for expansions and ChIP at INO1 and SPS2, and expansion rate analysis in yeast nuclease mutants. n, number of independent experiments; SEM, standard error of the mean; p values calculated by two-tailed Student's t test. (TIF) [file pbio.1001257.s012.tif]
